# Dr. John F. W. Whitbeck

**Published:** 1916-08

**Authors:** 


### OBITUARY

---

**Readers are requested to report promptly the death of all physicians in Western New York, or former residents of this region, or graduates of any medical school in Western New York, and to notify the families of the deceased of our desire to publish adequate Obituary notices.**

---

**Dr. John F. W. Whitbeck,** University of Pennsylvania 1870, died at his home in Rochester, N. Y., July 3, after a cardiac attack of three hours' duration. He had apparently been in excellent general health and went to his office as usual and then to another physician's office, where he first noticed symptoms of cardiac failure but was able to be taken home. He was a graduate of the University of Rochester in arts. He was born in Lima, N. Y. 72 years ago. After graduating in medicine, he studied in Germany and other European countries for three years, locating in Rochester in 1873 and specializing in surgery and gynaecology, though a conservative operator. His father practiced medicine in Rochester for over 40 years. Dr. Whitbeck was a member of the various local medical organizations and of those affiliated with the A. M. A., the American College of Surgeons, and others, and was for many years a member of the staff of the Rochester General (formerly City) Hospital, where as interne we had the opportunity to assist him in his work and to appreciate both his surgical and general medical skill and his kindly disposition. He was one of the kind of men who acquire success and recognition by leading rather than by pushing. He was largely instrumental in the establishment of Iola Sanatorium—a model institution for the relief of tuberculosis—and was President of the Board of Managers. He had served as President of the Rochester Academy of Medicine and of the Medical Society of the State of N. Y.

